# Lichenoid dermatosis after apalutamide treatment: Two case reports and literature review

**DOI:** 10.1016/j.jdcr.2026.02.035

**Published:** 2026-02-23

**Authors:** Chelsea Deitelzweig, Haya Raef, Nujood Alzahrani, Justin Cheeley

**Affiliations:** a Department of Dermatology, Emory University School of Medicine, Atlanta, Georgia; b Department of General Internal Medicine, Emory University School of Medicine, Atlanta, Georgia

**Keywords:** androgen receptor inhibitor, apalutamide, drug reaction, lichenoid, prostate cancer

## Introduction

Apalutamide is an oral selective inhibitor of the androgen receptor that is approved by the Food and Drug Administration for metastatic castration-sensitive and nonmetastatic castration-resistant prostate cancer.^1^ Apalutamide has been reported to produce dose-dependent maculopapular eruptions, generally occurring within 6 months of treatment, although the precise clinical and histopathologic presentation of lichenoid eruptions remains poorly characterized.^2^ As apalutamide use becomes more commonplace, understanding the spectrum and mechanisms of associated cutaneous reactions is important. Herein, we present 2 cases of lichenoid drug eruptions after apalutamide treatment and compare these findings with previously reported cases.

## Case presentations

### Case 1

**Fig 1:**
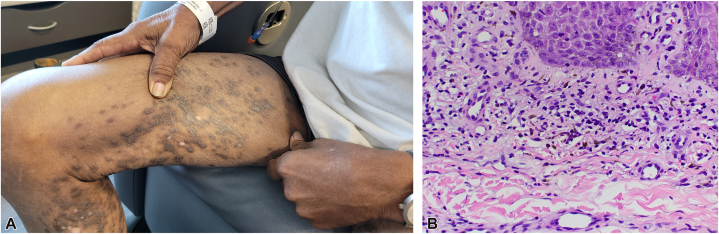
Apalutamide-related lichenoid reaction. **A,** Violaceous, scaly, flat-topped polygonal papules on the right lower extremity. **B,** Histopathology showing focal lichenoid epidermal interface alteration and a subjacent, superficial, band-like dermal lymphocytic inflammatory with scattered melanophages (hematoxylin-eosin stain; original magnification: 20×).

A 75-year-old African American male with a medical history notable only for stage IV prostate cancer metastatic to the bone and lymph nodes developed violaceous, scaly, flat-topped polygonal papules on his bilateral lower extremities, with sparse involvement on the arms, 6 months after initiating apalutamide 240 mg daily, a gonadotropin-releasing hormone antagonist daily, and denosumab monthly (Fig 1, *A*). There was no oral or genital involvement. He denied any associated pruritus or tenderness of affected areas. Given concern for drug-induced rash, apalutamide was discontinued within 1 month of onset by his urologist. All other medications, including denosumab, were continued.

Despite discontinuation of apalutamide, the rash persisted, and the patient was evaluated by the Dermatology team 1 month later during an inpatient stay. A punch biopsy was obtained from the right thigh, revealing focal superficial band-like dermal lymphocytic inflammatory infiltrate with scattered melanophages and rare eosinophils (Fig 1, *B*). These findings were consistent with lichenoid dermatitis, likely drug-induced, with apalutamide thought to be the culprit. The patient was prescribed clobetasol 0.05% ointment with gradual improvement. Apalutamide was exchanged for an alternative antiandrogen agent, enzalutamide.

### Case 2

**Fig 2:**
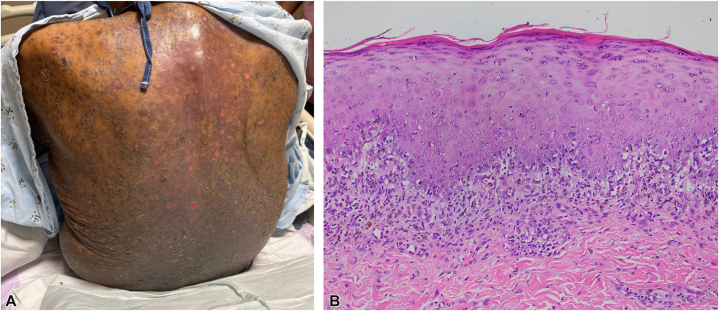
Apalutamide-related lichenoid reaction. **A,** Multiple exfoliative plaques involving the trunk and extremities. **B,** Histopathology showing epidermis with compact mild hyperorthokeratosis, hypergranulosis, and irregular acanthosis. There is dermal-epidermal interface alteration with dyskeratotic keratinocytes and a subjacent band-like lymphohistiocytic infiltrate (hematoxylin-eosin stain; original magnification: 20×).

An 86-year-old African American male with multiple comorbidities including atrial fibrillation (on warfarin), hypertension (on metoprolol tartrate, losartan, and amlodipine), hypercholesterolemia (on lovastatin), chronic kidney disease stage IV, gout (on allopurinol), and recurrent metastatic prostate cancer postradiation was initiated on apalutamide 240 mg daily, denosumab, and leuprolide acetate every 3 months. Two months after initiation, the patient was admitted for inpatient evaluation of a diffuse, pruritic eruption characterized by exfoliative plaques involving the trunk and extremities (Fig 2, *A*). There was oral involvement with erosions on the palate and buccal mucosa.

Two punch biopsies were obtained from the right thigh and abdomen. Histopathologic examination of both samples revealed lichenoid interface alteration along the dermal-epidermal junction with a band-like lymphocytic infiltrate and scattered eosinophils, consistent with a drug-induced lichenoid eruption (Fig 2, *B*). Apalutamide was discontinued and transitioned to another antiandrogen therapy, darolutamide. All other medications, including denosumab, were continued. The patient was treated with triamcinolone 0.1% cream resulting in gradual improvement.

## Discussion

Apalutamide is a selective androgen receptor inhibitor that disrupts androgen signaling to promote tumor cell death. Skin toxicity has been a recognized side effect since early clinical trials in the SPARTAN (A Study of Apalutamide (ARN-509) in Men With Non-Metastatic Castration-Resistant Prostate Cancer) and TITAN (A Study of Apalutamide (JNJ-56021927, ARN-509) Plus Androgen Deprivation Therapy (ADT) Versus ADT in Participants With mHSPC) studies.^1^ Reported dermatologic reactions ranged in severity and presentation, including maculopapular, urticarial, blistering, and exfoliative rashes. Both trials found that skin eruptions occurred more often in Japanese patients compared to other racial groups, attributed to lower average body weight.^2^ Similar trends have been observed in Chinese cohorts, with 1 study documenting skin reactions secondary to apalutamide in approximately one-third of patients,^3^ and more recent findings reporting incidence up to 50%.^4^

Further characterization of these dermatologic events has emerged through case reports, highlighting growing recognition of this skin reaction pattern.^5^ A total of 8 patients with biopsy-proven apalutamide-induced lichenoid interface dermatitis are summarized in Table I. Across our cases and the reviewed literature, the exanthem was typically widespread, most often involving the trunk and extremities, with mucosal involvement reported in only 2 cases. Clinical presentations of drug-induced lichenoid eruptions are variable, both among our patients and published cases, but tend to be more generalized than idiopathic lichen planus, occasionally showing eczematous or psoriasiform features and typically sparing mucosal and flexural areas. Histologically, these eruptions resemble idiopathic lichen planus with a dense band-like lymphocytic infiltrate in the dermis that obscures the dermoepidermal junction; findings observed in most of the reported cases, including our own.

**Table I:** Overview of apalutamide-induced lichenoid dermatosis reported in the literature

Publication	Patient age	Rash onset	Rash distribution & morphology	Mucosal involvement	Pathology	Treatment	Outcome
Guglielmini et al,^6^ Case Report, Italy	83	14 mo after treatment	Extensive lichenoid erythematous, atrophic, purplish/hyperpigmented rash on the trunk, arms, neck, buttocks, and legs	No	Epidermal hyperplasia with orthokeratosis, hypergranulosis, mild basal vacuolar degeneration, and necrotic keratinocytes. A band-like lymphocytic infiltrate containing eosinophils and melanophages was seen in the papillary dermis	Apalutamide discontinued, oral corticosteroid initiated	Pruritus and lesions improved within a few days
Class et al,^7^ Case Report, USA	72	2.5 mo after treatment	Widespread lichenified papules and plaques were noted on the face, chest, back, and legs	No	Lichenoid interface dermatitis with numerous eosinophils	Apalutamide discontinued, triamcinolone ointment initiated	The rash initially improved after 2 wk but re-emerged upon resuming apalutamide. Apalutamide was subsequently switched to enzalutamide, leading to a resolution of the rash after 2 mo
Cremante et al,^8^ Case Report, Italy	57	4 mo after treatment	Erythematous papules with hyperkeratotic scale involving 10% to 30% BSA	No	Hyperkeratosis, hypergranulosis, and acanthosis with a “saw tooth” appearance, with numerous Civatte bodies and lichenoid infiltrate	Apalutamide continued, topical steroids and oral antihistamine initiated	Rash gradually resolved
Tohyama et al,^9^ Case Report, Japan	80s	2 mo after treatment	Diffuse erythematous papular rash with slight scale	No	Hyperkeratosis, vacuolar degeneration, and civatte bodies. The perivascular area of the upper dermis infiltrated with lymphocytes and a small number of eosinophils	Apalutamide discontinued, topical clobetasol initiated	Rash gradually resolved after 1 mo of treatment
Tohyama et al,^9^ Case Report, Japan	70s	1.5 mo after treatment	Diffuse pruritic erythematous rash	No	Intraepidermal infiltration of lymphocytes, vacuolar degeneration of basal cells, civatte body, and eosinophil infiltration in the dermis	Apalutamide discontinued, topical clobetasol initiated	Rash resolved after 5 wk
Prontskus et al,^5^ Database Query, France	78	2 mo after treatment	Generalized skin rash	Yes (mouth)	Lichenoid dermatitis, with vascular proliferation, compatible with lichenoid toxidermia	Apalutamide discontinued, topical steroids initiated	Rash gradually resolved
Prontskus et al,^5^ Database Query, France	64	4 mo after treatment	Generalized pruritic maculopapular exanthema	Yes (mouth)	Lichenoid dermatitis	Apalutamide discontinued, topical steroids and oral antihistamine initiated	Rash persisted despite topical steroid and antihistamine initiation
Prontskus et al,^5^ Database Query, France	75	4 mo after treatment	Generalized pruritic erythematous rash with lichenification aspect	No	Lichenoid interface dermatitis, compatible with toxidermia.	Apalutamide discontinued, topical steroids initiated	Rash gradually resolved

Apalutamide dose for all patients, if reported, was 240 mg daily.

*BSA*, Body surface area.

Lichenoid drug eruptions typically have a delayed onset, potentially occurring up to 12 months after initiating therapy. In the context of apalutamide, reported cases demonstrated a time to onset ranging from 5 weeks to 14 months, with an average onset of approximately 4 months, consistent with another study.^5^ In most cases, symptoms improved or resolved following discontinuation of apalutamide and initiation of topical or systemic corticosteroids. However, 1 patient did not recover despite cessation and treatment, highlighting the potential for persistent or refractory reactions in some individuals.

Cutaneous reactions have been reported more frequently with apalutamide than with other antiandrogens, potentially due to a distinctive structural component of the drug. Ji et al proposed that the 2-cyanopyridine moiety in apalutamide can trigger an immune-mediated reaction.^10^ Prior studies demonstrated that this moiety exhibits higher reactivity than those in related compounds, which may explain why lichenoid eruptions are more associated with apalutamide than other androgen deprivation therapies, like enzalutamide.^10^ This process may underlie the development of lichenoid eruptions, although further studies are needed to better clarify the pathogenesis.

Apalutamide is the most likely trigger of the lichenoid eruptions in our cases, based on the timing of rash onset after drug initiation, rash morphology and pathology, improvement following discontinuation, and supportive evidence in the literature implicating apalutamide as a cause of drug-induced lichenoid dermatosis more strongly than the patients’ other medications, such as denosumab. Both patients shared a Naranjo Adverse Drug Reaction score of 4, indicating possible causation. Our findings add to the growing body of evidence that apalutamide can induce lichenoid eruptions among its diverse cutaneous toxicities. While skin-related adverse effects have been reported more frequently in Asian cohorts, with lichenoid cases previously reported in Japanese and Caucasian individuals, our patients were African American, emphasizing that such reactions can occur across diverse ethnic groups.

## Conflicts of interest

None disclosed.
